# Sudden Death Due to Traumatic Ascending Aortic Pseudoaneurysms Ruptured Into the Esophagus: 2 Case Reports

**DOI:** 10.1097/MD.0000000000000716

**Published:** 2015-04-17

**Authors:** Shixia He, Xiaorui Chen, Xiaowei Zhou, Qingqing Hu, Sunnassee Ananda, Shaohua Zhu

**Affiliations:** From the Department of Forensic Medicine (SH, XC, XZ, QH, SZ), Tongji Medical College, Huazhong University of Science and Technology, Wuhan, Hubei, China; and Ministry of Health and Quality of Life (SA), Mauritius.

## Abstract

We present 2 rare cases of patients with uncontrolled hemorrhagic shock induced by traumatic ascending aortic pseudoaneurysm rupture into the esophagus.

Two men were presented to the hospital after traffic accidents. Their chest radiograph showed no obvious signs of aortic damage or aortic pseudoaneurysms but only a small amount of high-density shadow in the mediastinum and no specific clinical signs besides chest tightness or chest tenderness. The first case was misdiagnosed as pulmonary contusion and pleural effusion, and the second case was misdiagnosed as mediastinal lesions in the mediastina. They were given symptomatic and supportive treatment. Unfortunately, they died suddenly after >1 month of traumatic accident.

At autopsy, ascending aortic pseudoaneurysms that broke into the esophagus and multiple organ hematocele were detected by gross examination. In histopathological examination, inflammatory cells and proliferated fibrous connective tissue were detected in the ascending aortic pseudoaneurysms, and the pathological gastrointestinal bleeding was not seen. The drugs and poisons were not found on toxicological analysis.

The 2 patients died as a result of hemorrhagic shock from traumatic ascending aortic pseudoaneurysm rupture into the esophagus. We suggest that thoracic surgeon should be aware of the possibility of aortic injury after chest trauma to reduce misdiagnosis and prevent similar accidents.

## INTRODUCTION

An aortic pseudoaneurysm can form following vascular injury and/or blunt chest trauma. It is caused by a defect in the inner layers of the vessels, the tunica intima and the tunica media, but usually with an intact tunica adventitia, or can involve all 3 layers, in which the blood flowed, and contained surrounding structures, although in true aneurysms all 3 layers remain intact.^1,2^ The morbidity and mortality of aortic pseudoaneurysms are historically >95% if the injury is left untreated. About 80% to 90% of all acute traumatic aortic injuries are immediately fatal.^3^ The overall mortality of thoracic aortic injuries that occur in the region of the aortic isthmus is >30% to 90%, and only 5% involves the ascending aorta.^4,5^

Common clinical signs and symptoms include local swelling with expansile pulsation and pain in the chest and back. Diagnosis of pseudoaneurysms with typical clinical manifestations is relatively easy, but nonobvious symptoms of pseudoaneurysms prone to misdiagnosis or miss. Radiological imaging, such as computed tomography (CT), computed tomography angiography (CTA), and magnetic resonance imaging (MRI), is quite sensitive to diagnose pseudoaneurysms. Pseudoaneurysms may rupture due to sudden increased pressure within the chest and abdomen.

Here, we report 2 cases of men who died >1 month after being discharged from the hospital where the aortic pseudoaneurysms were not detected. We also discuss the mechanism of ascending aortic pseudoaneurysm ruptured into the esophagus and other mediastinal adjacent structures in detail.

## CASE PRESENTATION

### Case 1

A 38-year-old male driver was involved in a road traffic accident. He was sent to the hospital 2 hours later with swelling of the right lower limbs and chest tightness. CT scan of the chest showed a small amount of high-density shadow in the mediastinum and a few lamellar high-density shadows in the right lung. The right femur was distally fractured. His diagnoses were as follows: open right distal femur fractures; the right lung contusion and bilateral pleural effusion; scalp hematoma; and multiple soft tissue contusion in the body. Open reduction and internal fixation was applied to right distal femur fracture. His vital signs were stable, cardiopulmonary and abdominal diagnoses showed no abnormalities at the time of discharge after 25 days anti-inflammatory and hemostasis treatment in the hospital. However, 2 months later, he died after repeated coughs.

Forensic autopsy was carried out 12 hours after death. External examination showed healing soft tissue injuries and operation scar in his left thigh and a comminuted fracture of the distal femur. No obvious abnormalities were seen on the head, neck, and chest.

**FIGURE 1:**
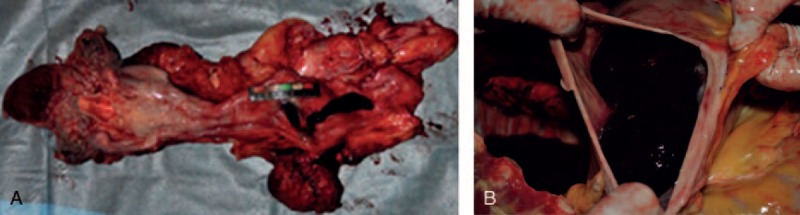
(A) Ascending aorta pseudoaneurysm ruptured into the esophagus and a fistula-like defect on the esophageal wall. (B) Clotted blood in the stomach.

On internal examination, a pseudoaneurysm, measuring 6 × 5 × 5 cm, was found 6 cm away from the aortic valve. The pseudoaneurysm was burst opened and adhered to the midsection of the esophagus where much blood clots were illustrated (Fig. 1A). Stomach was filled with 1800 g of clotted blood. The duodenum and small intestine contained 500 mL of liquid blood (Fig. 1B). Trachea and bilateral bronchial lumen had a small amount of blood. Other organs, including liver, gall bladder, spleen, stomach, and duodenum, were unremarkable.

**FIGURE 2:**
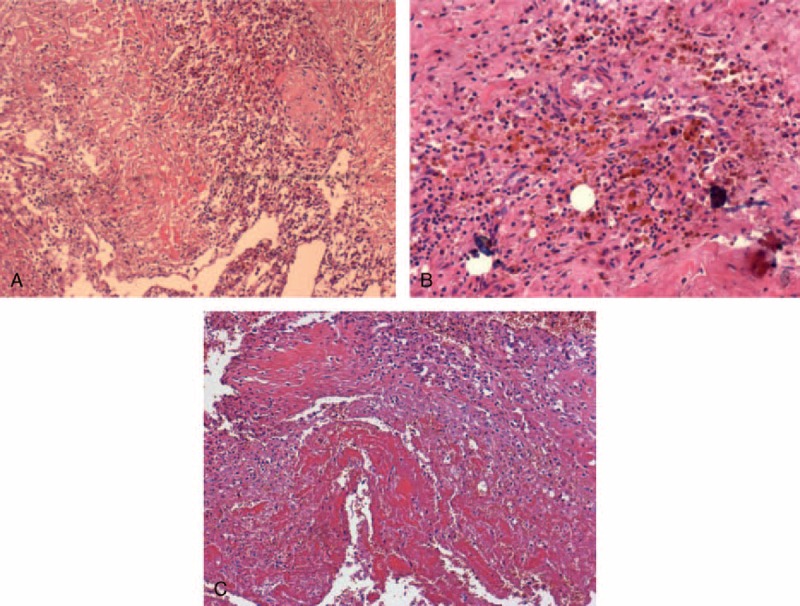
Microscopic view of pseudoaneurysm. (A) Collagen fiber necrosis and a large number of mononuclear infiltration of lymphocytes on the pseudoaneurysm of the ascending aorta wall (H&E ×100). (B) Hemosiderin cells in the aortic wall (H&E ×200). (C) Hemorrhage and necrosis in aortic pseudoaneurysm wall (H&E ×100). H&E = hematoxylin and eosin.

On histopathological examination, it was found that this ascending aorta pseudoaneurysm was made up of a large number of mixed intravascular agglutination and hyperplasia of fibrous connective tissue. A great number of red blood cells, monocytes, lymphocytes, and a small amount of neutrophils were observed in the pseudoaneurysm; hemosiderin deposits were also found in it (Fig. 2A–C).

Toxicological analysis done by gas chromatography–mass spectrometry (GC–MS) on blood, liver, and stomach excluded the presence of drugs and poisons. The cause of death was uncontrolled hemorrhagic shock induced by traumatic ascending aortic pseudoaneurysm rupture into the esophagus. The missed diagnosis gave rise to a medical negligence issue.

### Case 2

**FIGURE 3:**
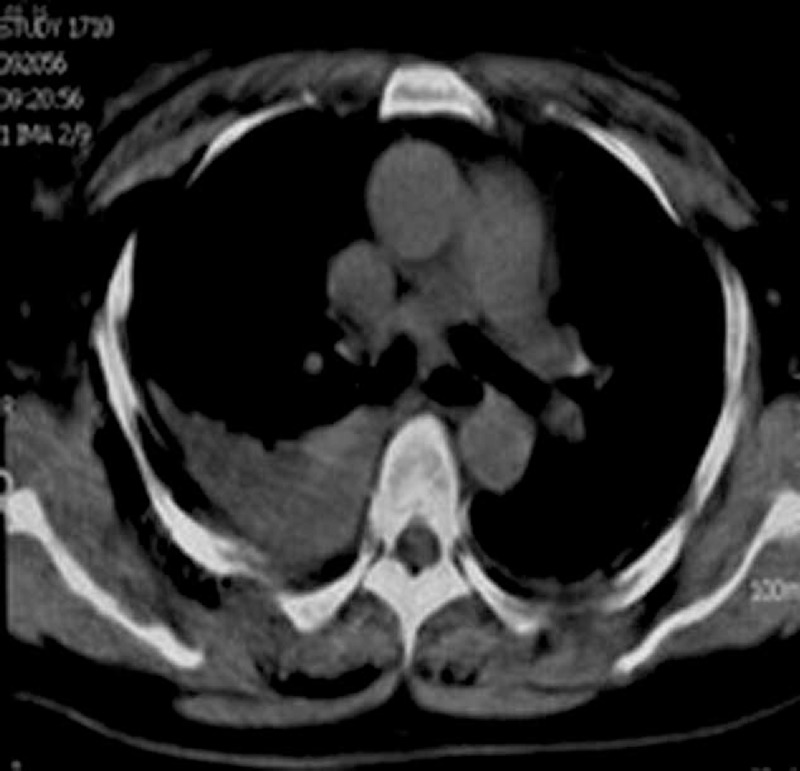
Mediastinal window of chest CT: bilateral patchy shadows, pleural effusion, and hemothorax.

A 50-year-old man presented to the emergency with multiple injuries after 30 minutes of a car accident. Physical examination showed blood pressure 100/60 mm Hg, and breast and abdominal tenderness. On the second day, chest CT demonstrated a 55-mm-thick overlapping shadow of semilunar soft tissue of posterior chest wall (Fig. 3), bilateral patchy shadows, and fractured eighth and tenth rib. The patient was drained 450 mL red bloody fluid on thoracic cavity with closed drainage 3 days later. His initial diagnosis revealed lung infections, posterior mediastinal space-occupying lesion. Because the patient had no further obvious discomforts, symptomatic and supportive treatment was implemented. However, 32 days after defecating, he felt discomfort, went into coma, poured a lot of blood from the mouth, resuscitation attempts failed, and he died.

Forensic autopsy was carried out the next day of his death. External examination demonstrated small pieces of abrasion scars on the right shoulder and the left lateral chest, which also had a drainage port. Internally, eighth and tenth rib was fractured with remote hemorrhage. A hematoma was wrapped by mediastinal tissue 20 cm away from the epiglottic cartilage measuring 13 × 10 × 4.0 cm. The ascending aorta had a breach of 1.5 cm in diameter through the aortic lumen, where a 3.5 × 1.5 cm broken hole adjacent to the esophageal wall was also found. The lumen of esophagus, stomach, duodenum, and upper jejunum was filled with a great deal of blood below the broken hole. The remaining organs were unremarkable.

Microscopic examination showed irregular thickening of the aorta, inflammatory cells infiltration under the tunica intima, and the fibrous connective tissue that proliferated and organized the hematoma walls. The other organs and tissues showed no significant abnormality. No evidence of drugs and poisons was found on blood, liver, and stomach on GC–MS. Death was attributed to massive hemorrhage of gastrointestinal (GI) tract due to ascending aortic pseudoaneurysm ruptured into the esophagus.

This study was approved by the Ethics Committee of Tongji Medical College, Huazhong University of Science and Technology, Wuhan, Hubei, China, and written informed consent was obtained from the family members of the 2 patients.

## DISCUSSION

In the aforementioned cases, brain, cardiac, and GI hemorrhage diseases could be excluded. In view of their death process, presence of blood accumulation in the esophagus, stomach, duodenum, and upper jejunum, and significant pathologies of histopathological examination, their causes of death were unambiguous. In retrospect, we can find some key factors throughout our 2 cases of deaths: aorta injury is a prerequisite for formation of pseudoaneurysms, its development is associated with misdiagnosis/missed and unresponsive medical behavior followed, thoracic and abdominal upheaval pressure is a incentive to induce pseudoaneurysms rupturing, and the ascending aortic pseudoaneurysms ruptured into the esophagus is determined by the close relationship of the pseudoaneurysms and esophagus.

Because of high-pressure aortic flowing, bleeding cannot easily be wrapped around the tissue; only 2% to 5% aortic damage develops into partial aneurysm or aortic pseudoaneurysms. More than 90% of aortic false aneurysms involve the aortic isthmus.^6–8^ The rough incidence of ascending aortic pseudoaneurysms are 10% to 14%.^9,10^ In our 2 cases, pseudoaneurysms all occurred in the ascending aorta, which was a rare site of pseudoaneurysms formation.

Aortic pseudoaneurysms are noble incidents that can occur secondary to trauma, infection, or as a complication of cardiac surgery^11,12^; chest and abdomen deceleration injury is the most common type of trauma. The exact mechanism of traumatic aortic injuries is complex; 2 popular theories include the “Whiplash” and the “osseous pinch” theory to reveal this phenomenon.^13–16^ Traumatic disruption of the aorta occurs when a number of stresses, generated by a large and sudden change in velocity, combine to tear the aortic wall usually immediately distal to the ligamentum arteriosum. At the time of admission, the deceased two owed the possibility of chest injuries after traffic accident with chest pain or tenderness.

Misdiagnosis or missed is one of the important factors of the whole event. Clinical presentations of pseudoaneurysms include local mass, severe chest pain, myocardial ischemia, and heart failure secondary to valvular regurgitation or cardiac compression. However, in the presented cases, there were no specific clinical signs besides chest tightness or chest tenderness. Catheter aortography remains the gold standard for assessing the aortic pseudoaneurysms. Other commonly used methods include chest x-ray, helical CT, 64-slice spiral CT, MRI, and color Doppler sonography. The specific imaging findings are as follows: on plain chest radiography and CT, there may be widened mediastinum and aortic junction blur. The arterial blood flow echo and the signal of the blood flow within a soft tissue mass on the Doppler sonography and MR, contrast material extravasation on the digital subtraction angiography, respectively.^17,18–21^ But the chest CT did not find these typical signs of aortic pseudoaneurysms in our 2 cases. The early incidence of pseudoaneurysms rupturing was up to 30%, and the mortality rate was 32% to 40%. Thoracotomy repair, prosthetic vessel replacement, and stent intervention can effectively prolong the survival time of the survivors; a manifest aortic tear can be liable detected and not missed or misdiagnosed. Since the diagnosis of error, the 2 patients did not receive timely and effective treatment, and hospitals should bear some responsibility for their death.

Misdiagnosis of aortic pseudoaneurysms is not uncommon worldwide. Bleeding after pseudoaneurysms rupture can penetrate the trachea, thoracic, mediastinal, lung, and other adjacent structures, thus leading to different degrees of hemoptysis, mediastinal hematoma, pleural or pericardial hemorrhage, and other complications.^16^ Fistula was diagnosed as the main reason for the above clinical manifestations. Pericardial tamponade, bronchiectasis, and lung cancer were often misdiagnosed as other pathogens for the aortic pseudoaneurysms. Our first case was misdiagnosed as pulmonary contusion and pleural effusion in mediastinal window, the second case was misdiagnosed as mediastinal lesions in the mediastina, or they all were missed diagnosis of pseudoaneurysms. Because of atypical clinical manifestations and imaging examinations in our 2 cases, the lessons of misdiagnosis are as follows: doctors lacked sufficient aortic aneurysm knowledge and poor appreciation of a history of blunt chest. Peradventure physicians ignored the possibility of chest injury due to excessive attention to other parts of the damage in these compound injury patients; and chest physicians did not conduct proper identification or appropriate diagnostic studies to exclude the pseudoaneurysms. No further imaging examinations were ruled out or confirmed the possibility by CTA and MRI. Therefore, for patients with chest injuries, in order to avoid similar accidents, these imaging chest examinations should be subject to review or periodic review.

Aortic pseudoaneurysms rarely broke into the esophagus as seen in our case. The position relationship between the esophagus and the ascending aortic pseudoaneurysms can explain this phenomenon: it is well known that large blood vessels, trachea, esophagus, thymus lymph tissue, and nerve all are in the mediastinum. The small cut of ascending aorta was easily wrapped by the middle esophagus, as in our 2 cases, when bleeding could be stopped temporarily. Meanwhile, gradually increasing pseudoaneurysms compressed the esophagus in ischemic necrosis, esophagus was increasingly resistant to the expansion of the pseudoaneurysms accordingly. In addition, the lower segment of the esophagus near the esophageal stenosis was mainly made up of the smooth muscle layer, which was thinner and lacks the expansion of buffer organization. Therefore, pseudoaneurysms and adjacent tissues have a similar antagonistic relationship.

Drastic changes of thoracic and abdominal pressure are predisposing factors of pseudoaneurysms ruptured into the esophagus: strong changes of blood and intraabdominal pressure can induce the false aneurysm rupturing. When the patient was in repeated coughing, abdominal pressure and arterial pressure increased drastically, and the sharply enlarged pseudoaneurysms oppressed and conflicted the necrotic esophageal into sudden rupturing, to cause the severe upper GI bleeding, even hemorrhagic shock. Pathological GI bleeding can be caused by a variety of clinical disease, such as decompensation stage of hepatocirrhosis, splenomegaly, gastric varices, hemorrhagic gastritis, esophagitis, and so on.^22^ In the presented case, the pathological GI bleeding were exclusion without specific findings on autopsy and histological examinations on those organs and tissues. With the above analysis, we can see the cause of death was the hemorrhagic shock aroused by ascending aorta pseudoaneurysms rupturing into the esophagus combined with the chest blunt trauma, the death process, and excluding poison damage effect in the 2 cases.

This report highlights the need for clinicians to recognize this rare type of ascending aortic pseudoaneurysms death; the purpose is to prevent the happening of similar deaths. At present, symptomatic pseudoaneurysms are easily diagnosed clinically. However, subclinical pseudoaneurysms, without typical clinical manifestations and radiographic signs of aortic injury, may be misdiagnosed/missed. These 2 examples suggest that thoracic surgeons should cautiously evaluate the possibility of pseudoaneurysms combining with a history of blunt chest, and closely observe the clinical manifestation and radiological changes; attention cannot be focused solely on signs and symptoms like our cases, and regular review of the aortic situation is required.
